# Saffron for mild cognitive impairment and dementia: a systematic review and meta-analysis of randomised clinical trials

**DOI:** 10.1186/s12906-020-03102-3

**Published:** 2020-11-09

**Authors:** Zahra Ayati, Guoyan Yang, Mohammad Hossein Ayati, Seyed Ahmad Emami, Dennis Chang

**Affiliations:** 1 Department of Traditional Pharmacy, School of Pharmacy, Mashhad University of Medical Sciences, Mashhad, Iran; 2 NICM Heath Research Institute, Western Sydney University, Westmead, NSW 2145 Australia; 3 School of Traditional Medicine, Tehran University of Medical Sciences, Tehran, Iran

**Keywords:** Dementia, Alzheimer’s disease, MCI, Saffron, Iridaceae, Systematic review

## Abstract

**Background:**

Saffron (stigma of *Crocus sativus* L.) from Iridaceae family is a well-known traditional herbal medicine that has been used for hundreds of years to treat several diseases such as depressive mood, cancer and cardiovascular disorders. Recently, anti-dementia property of saffron has been indicated. However, the effects of saffron for the management of dementia remain controversial. The aim of the present study is to explore the effectiveness and safety of saffron in treating mild cognitive impairment and dementia.

**Methods:**

An electronic database search of some major English and Chinese databases was conducted until 31st May 2019 to identify relevant randomised clinical trials (RCT). The primary outcome was cognitive function and the secondary outcomes included daily living function, global clinical assessment, quality of life (QoL), psychiatric assessment and safety. Rev-Man 5.3 software was applied to perform the meta-analyses.

**Results:**

A total of four RCTs were included in this review. The analysis revealed that saffron significantly improves cognitive function measured by the Alzheimer’s Disease Assessment Scale-cognitive subscale (ADAS-cog) and Clinical Dementia Rating Scale-Sums of Boxes (CDR-SB), compared to placebo groups. In addition, there was no significant difference between saffron and conventional medicine, as measured by cognitive scales such as ADAS-cog and CDR-SB. Saffron improved daily living function, but the changes were not statistically significant. No serious adverse events were reported in the included studies.

**Conclusions:**

Saffron may have the potential to improve cognitive function and activities of daily living in patients with Alzheimer’s disease and mild cognitive impairment (MCI). However, due to limited high-quality studies there is insufficient evidence to make any recommendations for clinical use. Further clinical trials on larger sample sizes are warranted to shed more light on its efficacy and safety.

## Background

Mild cognitive impairment (MCI) is defined by mild quantifiable decline in cognitive function greater than expected for an individual’s age and education level, but essentially preserved functional abilities [1–3]. It is regarded as a risk state for dementia. The estimated prevalence of MCI in population-based studies ranges from 10 to 20% in people older than 65 years of age. Currently, there is no proven effective medication in treatment of MCI [4].

Dementia is a major cognitive disorder which is characterized by any significant cognitive decline from a previously higher level of functioning and compromises social and/or occupational functions [5]. Alzheimer’s disease (AD) is the most common neurodegenerative disorder and the most prevalent cause of dementia worldwide with an estimated prevalence of 10–30% in those aged > 65 years [6–8]. It is characterized by cognitive decline with loss of memory [9]. No pharmaceutical medicines are currently available to cure dementia, although some medications such as memantine (N-methyl-D-aspartate (NMDA) channel blocker), and galantamine, donepezil and rivastigmine (cholinesterase inhibitors) are used clinically to manage symptoms of the disease. However, these medications have some side effects such as headache, confusion, nausea and vomiting and leg cramp which are reported to be intolerable in some patients [10, 11].

*Crocus sativus* (saffron) which belongs to the Iridaceae family has a long history of use as a spice, colouring agent and herbal medicine since ancient times. It has been suggested that saffron is effective for several diseases such as depression, respiratory and cardiovascular disorders [12]. Saffron has also been used in traditional Persian and Chinese medicines to restore and enhance memory [13–15]. Recent clinical trials [16–19] and reviews [20–32] revealed the positive effects of saffron on cognitive deterioration and improving functional and behavioural disturbances in patients with dementia and MCI. However, the efficacy of saffron on dementia and MCI is still unclear. Although saffron has been used as a food additive for many centuries implying its safety in human consumption, the toxicity and safety of saffron requires careful evaluation when used as a medicinal herb. Several clinical trials have directly evaluated the safety of saffron. Saffron with doses less than 1.5 g/daily is considered relatively safe in healthy humans, however, toxic effects are reported with doses 5 g/daily and above with a lethal dose of about 20 g/daily [31]. The safety of saffron in MCI and dementia needs to be further investigated given the reduced renal and hepatic functions in these elderly cohorts.

To the best of our knowledge, no systematic reviews are yet conducted to assess the efficacy and safety of saffron for MCI and dementia. Accordingly, we aimed to review the effectiveness and safety of saffron for the treatment of MCI and dementia systematically and perform a meta-analysis to assess the magnitude of these effects, when possible.

## Methods

This systematic review is registered in PROSPERO, International prospective register of systematic reviews (Registration number: CRD42019127560). We followed the Preferred Reporting Items for Systematic Reviews and Meta-Analysis Statement (PRISMA) guidelines [33] to ensure comprehensive and transparent reporting of methods and results.

### Literature search and study selection

Two authors (ZA and GYY) independently conducted literature search from Web of Science, Pub-Med, Scopus, EMBASE and Cochrane Library, and two authors (GYY and MHA) independently conducted literature search from four major Chinese databases including China Network Knowledge Infrastructure (CNKI), Chinese Scientific Journals Database (VIP), Sino-Med Database and Wan Fang Database, from their inception until 31st May, 2019.

The English searching terms included “*Crocus sativus*”, “Saffron”, “Croci Stigma”, “iridaceae”, “Zafran” and “Alzheimer*”, “mild cognitive impairment”, “senile dementia”, and “random*”. The Chinese searching terms included mild cognitive impairment (“*qing_du_ren_zhi_zhang_ai*” and “*qing_du_ren_zhi_gong_neng_zhang_ai*”), Alzheimer’s disease (“*a_er_ci_hai_mo*”, “*a_er_ci_hai_mo_bing*”, “*a_er_zi_hai_mo*”, “*a_er_zi_hai_mo_bing*”, “*lao_nian_chi_dai*”, “*a_er_cai_mu_shi*”, “*lao_nian_xing_chi_dai*” and “*lao_nian_qi_chi_dai*”), saffron (“*hong hua*”), and randomized (“*sui_ji*”).

Two authors (ZA and GYY) independently screened the potential titles and abstracts according to the inclusion and exclusion criteria. The full texts of potentially eligible articles were retrieved and independently assessed by two authors (ZA and GYY) for eligibility. Any discrepancies were identified and resolved through discussion with a third author (DC). To assure inclusion of all relevant papers, reference lists of primary extracted studies and review papers were independently hand searched by two reviewers.

### Inclusion and exclusion criteria

#### Study design

Randomised controlled trials (either parallel or cross-over designs), with at least one group involving saffron for the treatment of MCI or dementia (including Alzheimer’s disease, vascular disease, Lewy body dementia, mixed dementia, Parkinson’s disease related dementia, and frontotemporal dementia) regardless of severity were included. For cross-over trials, only the outcomes of the first period were included. Other types of human studies such as quasi-randomized trials were excluded.

#### Participants

Participants diagnosed with any one of the following criteria as mild cognitive impairment were included: (a) The Diagnostic and Statistical Manual of Mental Disorder (DSM) III, III-R or IV; (b) The International Classification of Disease (ICD) version 9 or 10; (c) Petersen criteria; (d) European Consortium on Alzheimer’s disease.

Participants diagnosed with any one of the following criteria as dementia were included, regardless of severity and disease course: (a) The Diagnostic and Statistical Manual of Mental Disorder (DSM) III, III-R or IV; (b) The International Classification of Disease (ICD) (9th or 10th edition); (c) The National Institute of Neurological and Communicative Disorder and Stroke-Alzheimer’s Disease and Related Disorder Association (NINCDS/ADRDA).

Participants diagnosed with any of the following criteria as Vascular dementia were included: (a) DSM-III, III-R or IV criteria for the diagnosis of vascular dementia (b) The National Institute of Neurological Disorders and Stroke (NINDS) and the Association Internationale pour la Recherche et l’Enseignement en Neurosciences (AIREN) criteria for the diagnosis of vascular dementia.

#### Interventions

Any forms of saffron (powder, extract, or oil) were included. Studies that examined saffron in combination with other ingredients and studies which evaluated the effect of an active component of saffron were excluded.

#### Control

Studies that compared saffron with placebo, no treatment and conventional treatments were included. Co-interventions were also allowed, if applied in all arms.

#### Outcome measures

The primary outcome was cognitive function. The secondary outcomes included activities of daily living (ADL), quality of life (QoL), global clinical assessment, daily living function, psychiatric assessment and safety.

### Data extraction

A standardised, pre-piloted form was used to extract data from the included studies for assessment of study quality and evidence synthesis. Data to be extracted were as follows: study population and baseline characteristics, interventions and control condition, dosage and duration of intervention, outcome measures, and main results.

If relevant information was not available in the paper, corresponding authors of the papers were contacted via email three times at reasonable intervals.

### Quality assessment

The risk of bias was evaluated independently by two authors (ZA and GYY) using the Cochrane collaboration recommended tool [34]. We assessed six biases accordingly, including: selection bias (random sequence generation and allocation concealment), performance bias and detection bias (blinding), attrition bias (incomplete outcome data), reporting bias (selective outcome reporting) and other bias. We categorised each item in to “low”, “unclear” or “high risk” of bias. If a trial met all criteria, a low risk of bias was given; if a trial met none of the criteria, a high risk of bias was given; and if a trial provided insufficient information to judge, unclear risk of bias was given. Any disagreement about the judgment of the risk of bias was discussed and resolved by involving a third author (DC).

### Statistical analysis

Meta-analyses were performed using RevMan 5.3 software. Data were summarised by odds ratios (OR) with 95% confidence intervals (Cl), and data for continuous outcomes were performed using mean difference (MD) with 95% CI. Heterogeneity among trials was detected using I-squared (*I*^2^) index. *I*^2^ values greater than 50% were regarded as high heterogeneity. Random-effects model was used to conduct the meta-analysis unless the *I*^2^ statistic was less than 25%. We did not perform funnel plots to detect publication bias because there were less than 10 trials under each outcome.

## Results

**Fig. 1:**
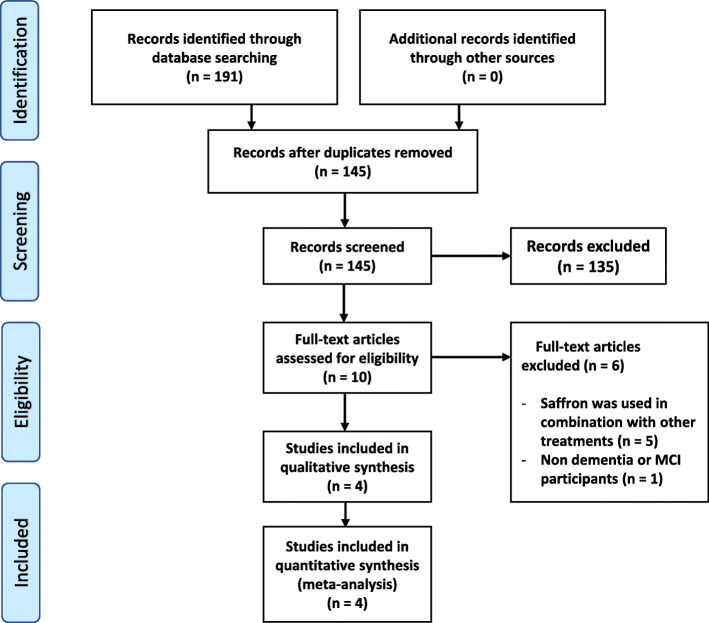
PRISMA flow diagram

The major English and Chinese databases were searched from their inception till 31st May 2019. The search returned 191 results which reduced to 145 after duplicates were removed. By screening the titles and abstracts for eligibility, a further 135 articles were excluded. After reviewing the full texts of the remaining 10 papers, 6 were further excluded for the following reasons: saffron was used in combination with other herbs or vitamins (*n* = 5) and saffron was evaluated on non-dementia or MCI patients (*n* = 1). Full details of search results are summarised in Fig. 1.

### Characteristics of the included studies

A total of 4 trials [16–19] with 203 patients were eligible for inclusion. All the included trials were 2-armed studies and published in English language. Three of the trials had been conducted in Iran and one in Greece. Among the 4 trials, 3 trials [16–18] focused on AD and the other trial [19] focused on MCI. No trial on other types of dementia was found.

The duration of treatments varied from 4 to 12 months. All the trials on AD used standard saffron extract 30 mg per day and the trial on MCI used saffron powder 125 mg daily. The comparison of saffron with placebo was performed in one trial [17], with conventional medicine in 2 trials [16, 18], and with no positive control or placebo in one trial [19]. Table 1 presents the detailed characteristics of the included trials.

**Table 1:** Characteristics of the included trials

Study ID	Subject	Mean age of Saffron group (year)	Mean age of control group (year)	Sample size, gender (F/M)	Study design	Form of saffron	Dosage (mg/day)	Control, mg/day	Duration (wk)	Outcome Measurement
Akhondzadeh 2010 [16]	AD	72.70 ± 6.20	73.85 ± 4.63	54 (25/29)	multicentre, double blind RCT	extract	30	donepezil, 10	22	ADAS-cog, CDR-SB, safety
Akhondzadeh 2010 [17]	AD	72.65 ± 3.89	73.13 ± 4.70	46 (21/25)	double blind RCT	extract	30	placebo	16	ADAS-cog, CDR-SB, safety
Farokhnia 2014 [18]	moderate to severe AD	77.73 ± 8.05	77.47 ± 7.99	68 (29/39)	double blind RCT	extract	30	memantine, 20	48	SCIRS, FAST, MMSE, safety
Tsolaki 2016 [19]	MCI	71.47 ± 6.73	69.72 ± 7.33	35 (26/9)	single blind, parallel RCT	powder	125	no treatment	48	MMSE, FRSSD, MoCA, NPI, GDS, MRI, EEG

**Notes:** *AD* Alzheimer’s disease; *MCI* Mild cognitive disorder; *ADAS-cog* Alzheimer’s Disease Assessment Scale-cognitive subscale; *CDR-SB* Clinical dementia rating scale-sums of boxes; *MMSE* Mini-Mental State Examination; *MoCA* Montreal Cognitive Assessment; *FRSSD* Functional Rating Scale for Symptoms of Dementia; *SCIRS* Severe Cognitive Impairment Rating Scale; *FAST* Functional Assessment Staging; *NPI* Neuropsychiatric Inventory; *GDS* Geriatric Depression Scale

### Risk of bias assessment

For random sequence generation, three trials [16–18] used computer-based randomisation and the remaining one just simply mentioned “randomized normal distribution” and did not report the specific method of random sequence generation.

Regarding the allocation concealment, 3 of the trials reported the allocation concealment method in detail [16–18] and the remaining one did not report any information about it. Three trials were double-blind trials (all on AD) and one was single blind (on MCI). Three trials (all on AD) described the number and reasons of withdrawal, while the remaining one did not report this information.

**Fig. 2:**
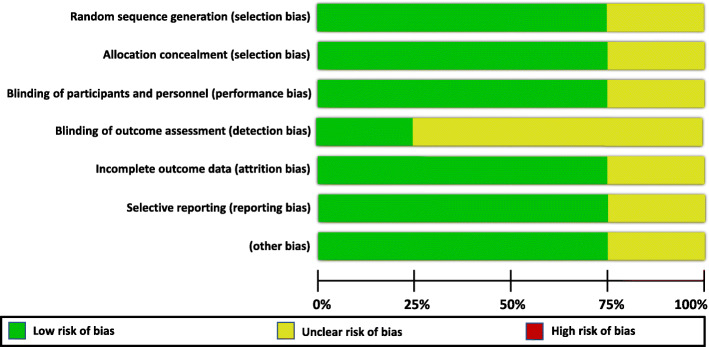
Risk of bias graph of the randomised controlled trials on saffron for mild cognitive impairment (MCI) and dementia

**Fig. 3:**
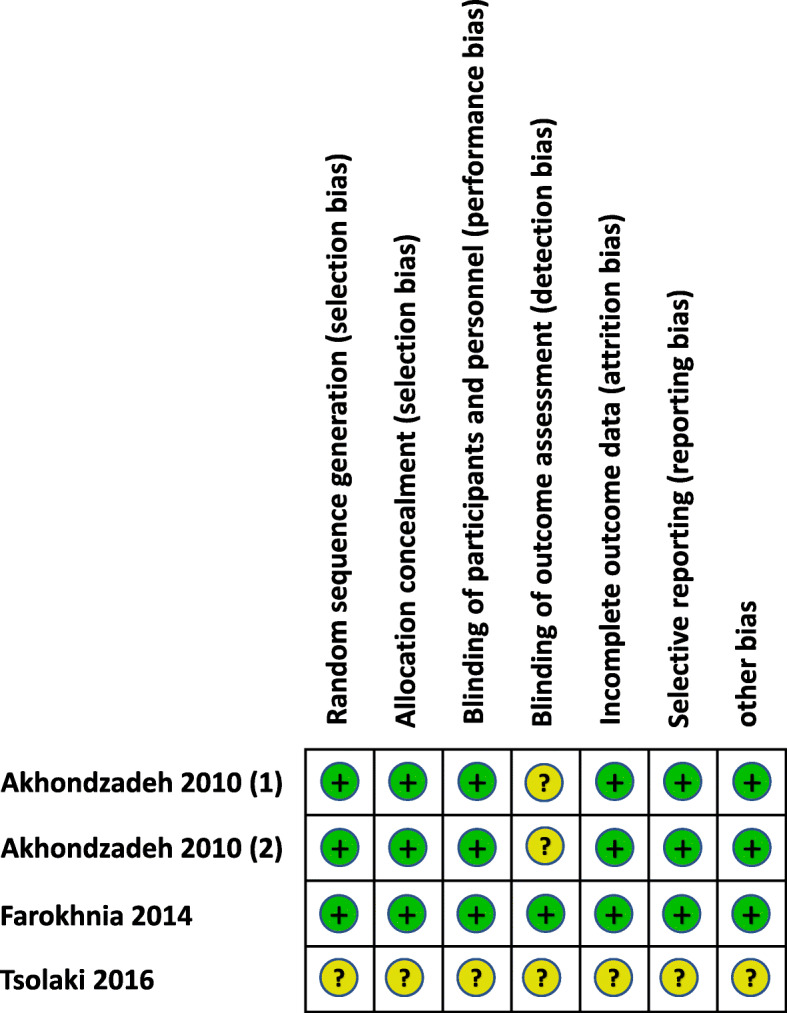
Risk of bias summary of the randomised controlled trials on saffron for mild cognitive impairment (MCI) and dementia

The general methodological quality of the trials of saffron for AD was moderate to high and for the trial of saffron on MCI was poor (Figs. 2 and 3).

### Findings from systematic review and meta-analysis

#### The effect of saffron on cognitive function

Three studies on AD reported this outcome [16–18]. Compared with placebo, one study by Akhondzadeh et al. [17] found that saffron improved ADAS-cog and CDR-SB scores significantly over 16 weeks. Compared with conventional medicine (donepezil), another study by Akhondzadeh et al. [16] demonstrated that saffron improved Alzheimer’s Disease Assessment Scale-cognitive subscale (ADAS-cog) and Clinical dementia rating scale-sums of boxes (CDR-SB) scores at 22 weeks, and the changes were statistically comparable between groups. Compared with conventional medicine (memantine), one study by Farokhnia et al. [18] revealed that saffron treatment caused an improvement in Mini-Mental State Examination (MMSE) and Severe Cognitive Impairment Rating Scale (SCIRS) scores at 48 weeks, and it was statistically comparable between groups (Table 2).

**Table 2:** Effect estimate for saffron on cognitive function, daily living function and psychological parameters

Outcome	Number of studies	Number of participants	Effect estimate by mean difference (IV, Random, 95% CI), ***p*** value	Study ID
**Cognitive function**				
ADAS-cog^1^	1	47	−0.19 [−2.28, 1.90], 0.86	Akhondzadeh 2010 [17]
ADAS-cog^2^	1	42	−7.77 [− 8.69, − 6.85], *p < 0.00001	Akhondzadeh 2010 [16]
CDR-SB^1^	1	47	0.06 [− 0.49, 0.61], 0.83	Akhondzadeh 2010 [17]
CDR-SB^2^	1	42	− 1.30 [− 1.52, − 1.08], *p < 0.00001	Akhondzadeh 2010 [16]
MMSE^a^	1	60	− 0.38 [− 1.12, 0.36], 0.32	Farokhnia 2014 [18]
MMSE^b^	1	35	1.07 [− 0.36, 2.50], 0.14	Tsolaki 2016 [19]
SCIRS	1	60	0.27 [− 0.36, 0.90], 0.40	Farokhnia 2014 [18]
MoCA	1	35	1.48 [−1.15, 4.11], 0.27	Tsolaki 2016 [19]
**Daily living function**				
FRSSD	1	35	− 1.13 [− 3.44, 1.18], 0.34	Tsolaki 2016 [19]
FAST	1	60	−0.03 [− 0.43, 0.37], 0.88	Farokhnia 2014 [18]
**Psychiatric assessment**				
NPI	1	35	−3.37 [−8.41, 1.67], 0.19	Tsolaki 2016 [19]
GDS	1	35	0.30 [−1.99, 2.59], 0.80	Tsolaki 2016 [19]

Note: *, statistically significant; 1, saffron compared with conventional medicine; 2, saffron compared with placebo; a, comparison of the changes from baseline to the end point between saffron and conventional group; b, comparison of the post scores between saffron and control group

One study on MCI also reported this outcome. Tsolaki et al. [19] found that saffron compared with “no treatment” was superior in improving cognitive function; the change in MMSE score in the saffron group was higher than that of the “no treatment” group. An improvement in Montreal Cognitive Assessment (MoCA) score was also observed in the saffron group compare to “no treatment” but the changes in MMSE and MoCA failed to reach statistical significance (Table 2). Additionally, the magnetic resonance imaging (MRI) results showed a small difference in the volume of left inferior temporal gyrus in favour of the saffron group [19].

#### The effect of saffron on daily living function

Daily living function in response to saffron treatment was evaluated in two studies [18, 19]. In the study on dementia by Farokhnia et al. [18], saffron treatment over 12 months improved Functional Assessment Staging (FAST) scale by − 0.03 when compared to conventional medicine (memantine) and no significant difference was found (Table 2).

Also, a 1-year saffron treatment reduced the score of Functional Rating Scale for Symptoms of Dementia (FRSSD) in MCI patients compare to no treatment (Tsolaki et al., [19]). However, this change was not statistically significant (Table 2).

#### Psychological assessment

In study by Tsolaki et al. [19], the Neuropsychiatric Inventory (NPI) score was improved after a one-year administration of saffron compare to the control group. In the same study, Geriatric Depression Scale (GDS) score was slightly higher at the end of trial in the saffron group when compared to that of control, but the changes in NPI and GDS are not statistically significant (Table 2).

#### Adverse events

Out of the 4 included trials, all 3 studies on AD reported safety information and the number of dropouts. One of the trials [17] which compared saffron with placebo reported one death in the control group due to myocardial infarction and mild adverse events such as dizziness, dry mouth, fatigue and nausea were reported in both saffron and control groups; no between group difference was found. In the other two trials [16, 18] that compared saffron with conventional medicine, mild adverse effects such as nausea, dry mouth and fatigue were reported and the differences between two groups were not statistically significant (Table 3). In one of these studies [16], one death was reported in the donepezil group due to myocardial infarction.

**Table 3:** Meta-analysis results for adverse events of saffron

Adverse events	Number of studies	Number of participants	Effect estimate (Odds Ratio (M-H, Random, 95% CI), ***p*** value
Nausea	3	156	0.90 [0.29, 2.80], 0.85
Dry mouth	3	156	1.53 [0.58, 4.07], 0.39
Fatigue	3	156	0.52 [0.17, 1.53], 0.23
Dizziness	3	156	0.51 [0.20, 1.31], 0.16
Confusion	1	60	1.00 [0.06, 16.76], 1
Agitation	1	60	0.19 [0.01, 4.06], 0.29
Sedation	1	60	0.31 [0.03, 3.17], 0.32
Vomiting	2	114	0.47 [0.04, 6.05], 0.56
Hypomania	2	96	3.990.42, 37.49], 0.23

Rates of patients remaining in the trials until the end were 90.47% for the saffron groups, 86.88% for conventional treatment groups and 86.95% for other control groups (placebo and no-treatment).

## Discussion

Saffron appears to be beneficial to cognitive performance in patients with MCI and AD. However, due to the small number of included clinical trials with diverse outcome measurements, we could not draw a definitive conclusion.

To the best of our knowledge, this is the first systematic review that examines the effects of saffron on cognitive performance in patients with MCI or dementia. No papers were identified to evaluate the effects of saffron on other types of dementia such as vascular dementia.

The general methodological quality of the three included trials on AD was high; Information on registration and the specific method of random sequence generation, allocation concealment and blinding were reported. However, the methodological quality of the only trial on MCI was poor due to the lack of information on the specific method of random sequence generation, allocation concealment, blinding, missing data and trial registration or protocol publication.

All included trials demonstrated that saffron had potential benefits in improving cognitive function for the treatment of AD and MCI. When measured by ADAS-cog and CDR-SB, treatments with saffron caused a clinically significant improvement compared with placebo. The magnitude of the changes in ADAS-cog appear to be high as it is in general agreement that a four-unit change on the ADAS-cog is required for a clinically significant/meaningful improvement [35]. In addition, compared with conventional medicine, the between group difference was not significant as measured by cognitive scales including ADAS-cog, CDR-SB and SCIRS.

The effect of saffron for daily living functioning is still in doubt. Although saffron treatment demonstrated a trend towards improvement in FAST and FRSSD, these changes were not significant.

Rates of retention and adherence of participants in AD trials were high in saffron groups and was slightly higher than that of the control groups. The safety profile of saffron appeared to be good. No serious adverse event (SAE) was reported in the saffron treatment groups. Mild adverse events such as nausea, fatigue, dry mouth and dizziness were observed in both intervention and control groups. However, the results show that the prevalence of the side effects observed between the intervention and control groups was not statistically significant.

The doses of saffron extracts used in the three included AD trials were the same (30 mg hydro alcoholic extract daily). The saffron extracts used in the AD clinical trials were standardised by safranal and crocin which are two of the most bioactive constituents of saffron. Each capsule contained 1.65–1.75 mg crocin and 0.13–0.15 mg safranal in all AD clinical trials. In the MCI trial, saffron powder (125 mg/day) was used and no information on the standardisation was provided. According to the previous studies, toxic dose of saffron powder is greater than 5 g/day [36, 37] and therefore the dose used in this study appears to be safe. However, the quality of saffron in the MCI trial is not clear.

The exact mechanisms underlying the effects of saffron on dementia remain unclear. However, transe-crocin-4, a main carotenoid constituent of saffron has shown to be able to inhibit A-beta fibrillogenesis formation [38]. A-beta fibrillogenesis is formed by oxidation of amyloid beta-peptide fibrils and plays a significant role in the pathophysiology of AD. Additionally saffron extract has been shown to possess a moderate inhibitory activity (30%) on acetyl-cholinesterase (AChE) and inhibits acetylcholine breakdown which is one of the main therapeutic targets for AD [39]. In an in vivo study, saffron also showed to increase antioxidant enzymes and decrease plasma levels of corticosterone suggesting that saffron is effective in improving the oxidative stress damage to the hippocampus followed by chronic stress [40].

In summary, only four trials were eligible to be included in this review using comprehensive search strategy both in English and Chinese databases with broad inclusion criteria. Although the methodological strength of the most included trials was reasonably strong, the number of participants in the included trials is low. A diverse outcome measures were used in these studies with different comparisons and it was not possible to conduct meta-analysis. Nevertheless, saffron has been shown to significantly improve ADAS-cog and CDR-SB scores in AD patients compared to placebo and was comparable to conventional medicine in improving cognitive function. However, given the small number of included trials, the clinical significance of these findings is in doubt.

## Conclusion

The results of this review suggest that saffron may be beneficial to improve cognitive function in patients with MCI and AD. No evidence was found to support the effects of saffron on other types of dementia. More high-quality randomised placebo-control trials are needed to further confirm the efficacy and safety of saffron for MCI and dementia.

## Data Availability

The datasets used and analysed during the current study are available from the corresponding author on reasonable request.

## Abbreviations

AD: Alzheimer’s disease
AChE: Acetyl-cholinesterase
ADAS-cog: Alzheimer’s disease assessment scale-cognitive subscale
ADL: Activities of daily living
AIREN: Association internationale pour la recherche et l’enseignement en neurosciences
CDR-SB: Clinical dementia rating scale-sums of boxes
Cl: Confidence intervals
CNKI: China network knowledge infrastructure
DSM: Diagnostic and statistical manual of mental disorder
FAST: Functional assessment staging
FRSSD: Functional rating scale for symptoms of dementia
GDS: Geriatric depression scale
ICD: International classification of disease
MCI: Mild cognitive impairment
MRI: Magnetic resonance imaging
MD: Mean difference
NINCDS/ADRDA: National institute of neurological and communicative disorder and stroke-alzheimer’s disease and related disorder association
NINDS: National institute of neurological disorders and stroke
NPI: Neuropsychiatric inventory
PRISMA: Preferred reporting items for systematic reviews and meta-analysis statement
PROSPERO: International prospective register of systematic reviews
QoL: Quality of life
SAE: Severe adverse event
